# Exploring implementation of a nationwide requirement to increase physical activity in the curriculum in Danish public schools: a mixed methods study

**DOI:** 10.1186/s12889-021-12152-2

**Published:** 2021-11-11

**Authors:** Sofie Koch, Charlotte Skau Pawlowski, Thomas Skovgaard, Natascha Holbæk Pedersen, Jens Troelsen

**Affiliations:** 1 Research Unit for Active Living, Department of Sports Science and Clinical Biomechanics, University of Southern Denmark, Campusvej 55, 5230 Odense M, Denmark; 2 Research and Implementation Centre for Human Movement and Learning, Department of Sports Science and Clinical Biomechanics, University of Southern Denmark, Campusvej 55, 5230 Odense M, Denmark; 3 Centre of Research in Childhood Health, Department of Sports Science and Clinical Biomechanics, University of Southern Denmark, Campusvej, 55 Odense M, Denmark; 4 Research Unit for Exercise Epidemiology, Department of Sports Science and Clinical Biomechanics, University of Southern Denmark, Campusvej, 55 Odense M, Denmark

**Keywords:** Physical activity, Implementation, School setting, RE-AIM, Mixed methods

## Abstract

**Background:**

In 2014, the Danish Government introduced a wide-ranging school reform that applied to all public schools in Denmark. A distinctive feature of the reform was that it became mandatory to implement an average of 45 min of daily physical activity within the curriculum. Using the RE-AIM framework as an evaluation tool, the objective of the current study was to evaluate the reach, effectiveness, adoption, implementation, and maintenance of mandatory physical activity within the curriculum at ten Danish schools.

**Methods:**

A complementary mixed-methods approach using accelerometers, questionnaires, and semi-structured interviews was conducted. A total of 10 schools were invited to participate, including 846 students, 76 teachers, and 10 school managers on various levels. Students were invited to wear an accelerometer for seven consecutive days. Teachers were invited to participate in a questionnaire, and school managers were encouraged to take part in a semi-structured interview.

**Results:**

Results showed that, on average, 45.2% of the students were active at least 45 min daily within the curriculum. Teacher and school management interest in physical activity, competencies development, and shared decision-making were identified as central factors for adoption of the requirement. Scheduling physical activity within scheduels and collaborations with external parties were found to influence implementation. Finally, internal coordination, motivated school staff, and school management priority were identified as central factors for maintenance.

**Conclusions:**

This study provides an evaluation on a nationwide physical activity requirement in Danish public schools. When introducing a wide-ranging nation-wide requirement on physical activity within the curriculum, school managers need to prioritize and support the implementation process. Teachers need to be involved in the decision processes in order to ensure motivation and local ownership. The study also highlights the benefits of an internal coordinator as well as development of a shared strategy among schools, municipalities, and other stakeholders in order to succeed with the implementation.

## Background

Physical activity (PA) is well-known for many health benefits [1]. The association between PA and academic achievement has further been given considerable attention. The applicability of PA to improve academic achievement is promising, but findings are mixed [2, 3]. However, more than eight out of ten adolescents are insufficiently physically active and PA levels among adolescents decrease significantly [4]. Strategies to counter this negative development are essential.

Schools are considered key settings for the promotion of PA in children as they provide convenient access to the majority of young people and feature core facilities, personnel and ethos to engage children in PA [5]. Therefore, both local, regional, and national governments and international bodies have released guidelines or policies mandating structured PA in schools [6, 7].

A wide-ranging school reform was introduced by the Danish Government in 2014 [8]. The school reform applied to all 1095 public schools in Denmark, and the overall aim was to ensure that all children met their full learning potential [9]. As part of the school reform, a requirement for all public schools to implement an average of 45 min PA within the curriculum per day was included for the first time in history. The Danish school reform is one of a select few examples worldwide of a scaled-up requirement mandating daily PA to be integrated into the school curriculum. Ideally, developing guidelines or policies for schools is, among other things, focused on translating evidence into community practice [10]. However, research suggests that most schools fail to implement PA policies at scale [11]. Translating and disseminating health-related policies into a real-world context is often challenging. Various barriers have previously been identified to affect the implementation of PA programs, such as lack of time, school management buy-in, and lack of facilities [11, 12]. However, limited research has been conducted on scaled-up real-world school-based PA programs, which call for a better understanding of the complex systems of contextual factors and practical implications driving both policy development and implementation in real-world contexts [13–15]. Thus, it is highly relevant to take a thorough look at the implementation of the nationwide PA requirement of the Danish school reform. The Reach, Effectiveness, Adoption, Implementation, and Maintenance framework (RE-AIM) has been deemed useful to evaluate internal and external validity of PA promotion programs, helping to provide a comprehensive evaluation [16, 17]. In particular, the RE-AIM framework has been used to evaluate real-world programs focusing on the implementation of PA in a school context [18–20]. Using the RE-AIM framework as an evaluation tool, the objective of the current study was to explore the reach, effectiveness, adoption, implementation, and maintenance of mandatory PA within the curriculum at ten Danish schools.

## Methods

### School context

In Denmark, public (state) schools are government (tax) funded and free of charge for all children between 6 and 16 years of age and mandatory unless attending private schools or homeschooling. The majority of children (77%) in Denmark attend public schools [8]. Most of the remaining children attend private or Danish free schools. Schools are typically organized in three tiers: pre-preparatory classes (grades 0–3, 5–9 years old), intermediate classes (grades 4–6, 9–12 years old), and lower secondary classes (grades 7–9, 12–15 years old). Children attend school 30–35 h per week, of which approximately 60–75 min per day are dedicated to recess. As a mandate of the school reform, daily PA was required to be integrated within the academic curriculum — within lessons or active breaks between lessons. The PA requirement also demanded that students had at least 60–90 min of physical education (PE) per week depending on age group. PE was included as part of the 45 min of daily PA, whereas recess was not [9]. The Government made no requirements on how to implement the mandatory PA components.

### Study design

The present study is part of a larger study, the Physical Activity in Schools After the Reform (PHASAR) study, aiming to evaluate the implementation and effects of the nationwide school-based PA legislation [21]. For this sub-study, a complementary mixed-methods design [22] was used to ensure a comprehensive understanding of the school level reach, effectiveness, adoption, implementation, and maintenance of the mandatory PA components.

### Study population

**Fig. 1:**
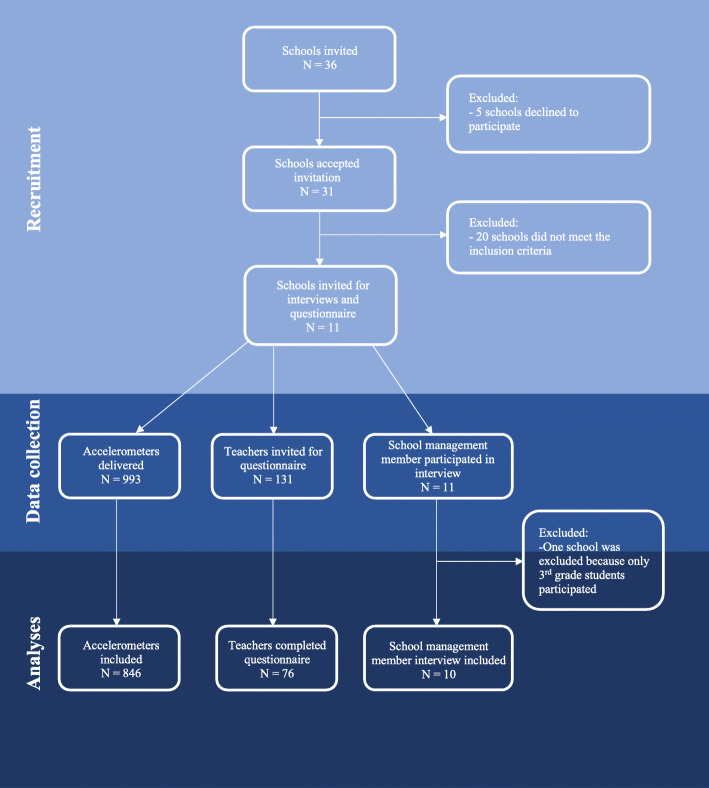
Flowchart of recruitment and measures

The ten schools included in the present study were recruited from the PHASAR study [21]. A total of 31 representative schools were included in the PHASAR study. The schools varied in geographic location, school size, municipal expenses per student, and disposable household income. School managers from eleven of the 31 schools were invited to participate in a semi-structured interview, excluding 20 schools for participation in this sub-study. From these eleven schools, ten schools that served adolescents 10–16 years old were included in the analysis. Thus, one school was further excluded, because they only served children 6–9 years old (see Fig. 1). Exclusion of children aged 6–9 years (grades 1–4) were chosen, as the PA level among the youngest children vary a lot from the PA level of adolescents. Maximum variation was used in selecting the schools to ensure a broad representation of school contexts, including geographic location, school size, and disposable household income. Characteristics of the ten selected schools are presented in Table 1.

**Table 1:** Characteristics of the ten participating schools

School	Geographic location	School size (number of students)	Level of school	Municipal expenses per student (USD)	Disposable household (USD)
1	Region of Southern Denmark	385	Grades 4–9	11,951	67,545
2	Region of Southern Denmark	324	Grades 0–6	11,951	49,789
3	Region of Southern Denmark	609	Grades 0–9	10,287	105,548
4	Region of Southern Denmark	359	Grades 0–9	10,287	68,798
5	Region of Southern Denmark	347	Grades 0–9	10,287	83,993
6	Region of Southern Denmark	866	Grades 0–9	11,129	69,559
7	Region of Southern Denmark	764	Grades 0–10	11,263	76,568
8	Region of Southern Denmark	605	Grades 0–10	11,052	85,112
9	Capital region	904	Grades 0–9	11,130	50,540
10	Capital region	580	Grades 0–10	11,130	49,367

The study population of interest were students in grades 5–9, who were asked to wear an accelerometer; teachers, who were asked to participate in a questionnaire; and one school manager from each school to participate in a semi-structured interview. Danish, Mathematics, and English courses take up a little more than half (53%) of the total teaching time in Danish public schools. Thus, teachers were eligible to participate in this study if they taught Danish, Mathematics, or English in one of the participating classes. The principals were recruited through purposeful sampling [23] to ensure knowledge from key respondents having insight into the implementation process of the PA requirement. The ten school managers consisted of three principals, four deputy principals, and three leading teachers with school management responsibilities.

### Re-aim

The RE-AIM framework was used to guide the evaluation, as it has shown useful when evaluating real-world programs and has a specific focus on implementation of new practices in a school setting [19, 20]. The definition, outcomes measures, and data sources of each dimension are presented below.

#### Reach

The reach dimension was defined as the characteristics of the ten schools selected for participation. Reach was described to ensure variation in relation to schools that were included in the present study compared to the non-participating schools (*n* = 21). School characteristics were assessed using data on geographic location, municipal expenses per student, school size, and disposable household income. School characteristics were assessed using the Danish Database of National Statistics.

#### Effectiveness

A more detailed effectiveness evaluation of the PHASAR study is described elsewhere [21]. In this sub-study, effectiveness was defined as *the percentage of students who, on average, reached 45 min daily PA within the curriculum*. The effectiveness dimension further reports on average minutes of PA within the curriculum and range in minutes of daily PA across schools. The students’ PA was objectively measured using accelerometers and compared across schools. In the present study, PA was defined as *standing with movement, walking, and running*. The definition was based on recent research, identifying sitting, standing, walking, running, and biking using accelerometers [24].

#### Adoption

Although all public schools in Denmark were required to implement mandatory PA within the curriculum, there was no guarantee that school managers and teachers would and/or could adopt this. The adoption dimension reports on the schools’ commitment to the mandatory PA within the curriculum and factors influencing adoption. Adoption rates were measured through a teacher questionnaire, whereas factors central for adoption were measured through semi-structured interviews with school managers.

#### Implementation

Implementation was defined as schools’ PA initiatives (e.g., structuring PA within schedules) and reports on the extent of PA delivered within the curriculum and schools’ process of implementing the mandatory PA within the curriculum. Furthermore, the dimension reports on factors influencing implementation of the mandatory PA within the curriculum. The extent of PA delivered within the curriculum was measured through a teacher questionnaire and factors central for implementation were measured through semi-structured interviews with school managers.

#### Maintenance

During the data collection, conducted 3–4 years after the introduction of the reform, most schools indicated that implementation had started, but had not been fully completed. Thus, the maintenance dimension reports on the extent to which schools have considered how to ensure maintenance. Maintenance was measured through semi-structured interviews within school managers.

### Data sources

**Fig. 2:**
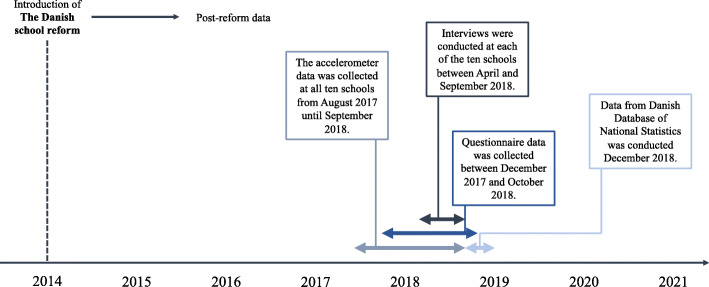
Timeline for data collection

Four data sources were collected: national statistics, accelerometers, a questionnaire aimed at teachers, and semi-structured interviews with school managers. Figure 2 presents a timeline of when each data source was collected.

#### Danish database of national statistics

The Danish Database of National Statistics was used to gather information on municipal expenses per student, school size, and disposable household income. The information was gathered in December 2018.

#### Accelerometers

The Axivity AX3 data logger was used to assess objectively measured PA. Students were invited to wear an accelerometer for seven consecutive days. Mounted in a belt placed directly against the skin around the subject’s right front thigh, the accelerometer provided an opportunity to calculate the duration spent on specific activity types. In this method, the acceleration is utilized in relation to the orientation of the subject’s thigh, which enabled us to distinguish very accurately between, for example, sitting and standing position. The accelerometer data was collected from August 2017 until September 2018. Standardized testing protocols were made to ensure data quality, and trained research assistants collected all data. Prior to the initiation of the study, a pilot study was conducted to optimize all study procedures. Accelerometry-based PA measures were analyzed for the curricular time during the school day (excluding recess).

#### Questionnaire

A questionnaire was designed to measure how often teachers employed PA within the curriculum and their attitudes towards the requirement (e.g., how often do you implement PA within the curricular teaching?; to what extent do you agree that PA could advance student learning?).

Several steps were taken to heighten the content validity of the questionnaire. Initially, the questionnaire was developed by two authors of the present study. Subsequently, the questionnaire was tested and discussed by several members of the PHASAR research group before pilot testing. The online procedure and the questionnaire were pilot tested with a group of teachers not included in the study to ensure face validity.

The questionnaire was designed and collected through the worldwide system Research Electronic Data Capture (RedCap). The use of electronic questionnaires made it possible to activate additional questions on specific answers, thereby ensuring that participants did not receive irrelevant questions. At the end of most questions, teachers were given the opportunity to add additional comments. The questionnaire was administered electronically, and the participating teachers were emailed a hyperlink to the questionnaire. Reminders were e-mailed to participants who did not respond (three times with 1 week between each reminder). Questionnaire data was collected between December 2017 and October 2018.

#### Semi-structured interviews

Ten interviews with school managers were conducted (one from each school). The interview guide was based on the Practical, Robust Implementation and Sustainability Model (PRISM) [25]. In particular, the interview focused on the adoption (e.g., how does the requirement fit within the existing school structure?), implementation (e.g., how do you organize your PA initiatives?), and maintenance dimensions (e.g., have you considered how to ensure maintenance of your initiatives?).

Interviews were conducted during a one-day visit to each of the ten schools between April and September 2018. All interviews were conducted one-on-one by the lead author and lasted between 25 and 60 min. Verbal consent was obtained from each participant to audio record the interview.

### Data analysis

#### Quantitative data analysis

STATA 16 (College Station, TX) was used to handle the quantitative data. Descriptive statistics were produced for the accelerometers’ data in order to gather information on the percentage of students who on average reached 45 min of daily PA. Students with non-wear or with less than one valid school day were excluded from the analysis. The activity types were analyzed in two-second intervals [24]. On this basis, PA was defined as the sum of the activity types stand with movement, walking, and running. Thus, sitting and standing were excluded during data processing.

Due to the paucity of responses in the questionnaire (*n* = 76), using a Likert scale became meaningless. Thus, 5-point Likert scale values were collapsed into two categories: “agree” and “disagree”, and 7-point Likert scale values were collapsed into four categories: “every day”, “weekly”, “monthly”, and “yearly or newer”. Descriptive statistics were produced on questionnaire data as well as data from the Danish Database of National Statistics. To analyze the reach component, two-sample t-tests were used to investigate the potential difference between participating and non-participating schools on municipal expenses per student, school size, and disposable household income. The level of significance was set at *p* < .05.

#### Qualitative data analysis

The interviews were transcribed by the lead author to ensure consistency. All interviews were transcribed verbatim directly into NVivo. Data was analyzed using a three-step qualitative thematic analysis [26]. First, all interviews were read through by two authors (SK and CSP) to ensure data familiarization. Coding was then conducted by both authors separately by marking all phrases concerning adoption, implementation, and maintenance, respectively. Secondly, all codes within the adoption, implementation, and maintenance dimension were read through. An open coding was then conducted, letting the data speak for itself [27]. As a result, themes were developed within the adoption, implementation, and maintenance dimension, respectively. Lastly, the findings were discussed among the two authors. Any discrepancies were resolved by consensus between the two authors [28].

### Ethical considerations

Prior to the data collection, students and their parents or guardians received information about the study. Consent took form as an oral and written informed passive consent from parents or guardians and students, entailing that all students were included in the study unless parents, guardians or the student decided to withdraw, which they were able to do at any time. Written consent was obtained from all principals, deputy principals, and leading teachers participating in the semi-structured interview. They were informed that they could withdraw from the study at any time. Schools and participants were anonymized by giving the schools numbers and naming participants by profession. The study was notified and approved by the Danish Data Protection Agency (2015-57-0008), who also gave legal advice and confirmed the legal basis of the informed passive consent.

## Results

### Reach

Denmark consists of five regions and despite the criteria of variation in geographical location in the school selection process, eight schools were located in the region of Southern Denmark and two schools were located in the Capital region. Thus, schools in the region of Southern Denmark were overrepresented in the present study. There was no significant difference in municipal expenses per student (*p* > 0.98), school size (*p* > 0.15), or disposable household income (*p* > 0.57) between participating and non-participating schools.

### Effectiveness

A total of 846 students were included in the analysis: 475 girls and 371 boys. On average, almost half of the participating students (45.2%) were active at least 45 min daily within the curriculum and thus reached the reform’s PA requirement. Large differences were, however, observed between schools. At the school with the lowest effectiveness (school #3), only 4.5% of students reached 45 min daily PA within the curriculum, while 82.6% of students at the school with the highest effectiveness (school #2) reached the requirement. Students were, on average, active 48.5 min daily within the curriculum. Large differences were seen between students, ranging from 5.2 min to 115.4 min of daily PA within the curriculum. An overview of accelerometer results is presented in Table 2.

**Table 2:** Accelerometers results on school level

School	Total	1	2	3	4	5	6	7	8	9	10
Grade	5–9	5–6	5–6	9	9	9	5–8	5–8	5–8	6–8	5–8
% students reaching 45 min. Daily PA within the curriculum	45.2	80.8	82.6	4.5	35.3	33.3	32.3	40.7	73.6	30.4	38.8
Average daily PA within the curriculum (minutes)	48.5	56.1	60.1	32.3	38.5	39.2	40.7	41.9	57.3	42.8	42.5
Range (min/max) of PA within the curriculum (minutes)	5.2–115.4	15.3–84.8	28.4–102.5	10.7–45.3	11.3–68.0	16.7–56.6	5.2–76.0	11.5–103.1	13.7–115.4	22.8–95.9	18.7–76.7

#### Adoption

Results from the teacher questionnaire (n = 76) revealed a general commitment to the mandatory PA component with 94.3% of all participating teachers believing that daily PA within the curriculum was important. In addition, 90.3% agreed that PA could advance student learning and 69.0% generally acknowledged that including PA in the curriculum activities had positive impacts.

From the interviews, four key-findings for adoption of the mandatory PA components were found: teacher and school management interest in PA, school management support, competencies development, and shared decision-making. All ten school managers interviewed found the mandatory PA within the curriculum meaningful, and at four schools (school #1, #2, #8, and #9) PA was already a central part of school culture before the 2014 reform. During interviews, however, school managers generally stated that some teachers were skeptical about the mandatory PA within the curriculum:

*“I think it depends on your interests. There are some [teachers, Ed.] who absolutely do not believe that PA does any good. That it’s rather a disturbing element”.* (Leading teacher, school #3)

The individual teacher’s interest in PA seemed crucial for commitment to the mandatory PA within the curriculum. Also, managerial support was important in order to take responsibility for developing a school culture supporting the delivery of PA within the curriculum.

*“It’s about making them [teachers, Ed. ] think it’s a good idea. And to do so, I need to be dedicated to it. And I need to take responsibility for the process in order to make it grow”.* (Deputy principal, school #6)

Furthermore, most school managers had experienced teachers who, from the outset, were poorly prepared for handling PA within the curriculum, challenging the adoption of the PA requirement. At five of the included schools (school #1, #2, #3, #4, and #9), it was therefore prioritized that all teachers participated in either a course or a workshop to strengthen their skills in how to include PA within the curriculum. The interviews showed that especially workshops or courses conducted by internal coordinators were useful for school adoption of the requirement, allowing teachers to continuously develop their competencies through ongoing follow-up workshops or courses.

*“It is our teachers who planned the pedagogical day, which means that after these workshops teachers know from whom they can be aided or take inspiration (…) Instead of hiring an external course organizer, who will leave afterwards along with the information. This way the information stays at the school, so that we continuously can get hold of it, which motivates teachers to take active part in it”*. (Head of school #3)

Delivering PA within the curriculum required that some teachers renewed learning formats — challenging their professional identity and calling for new or, at least, adjusted approaches to teaching. Some teachers believed a heightened focus on PA threatened the academic standard of their teaching:

*“Renewing your teaching is a really, really huge challenge. (...) It’s difficult because it’s a change of so many habits and working methods, which you [teachers, Ed.] believe students will learn a lot from”.* (Principal, school #3)

#### Implementation

Results from the teacher questionnaire showed that 9.5% of the participating teachers delivered PA within the curriculum on a daily basis, whereas 53.4% delivered PA within the curriculum on a weekly basis. Results showed a general consistency between schools. From the interviews, two key findings for implementation of PA within the curriculum were found: scheduling PA within schedules and collaborations with external parties. Six schools had scheduled PA within the school day to ensure that PA was delivered on fixed timepoints throughout the week. At three of six schools (school #4, #7, and #8) PA was scheduled as short daily lessons of 15–30 min dedicated to PA. The other three schools (school #1, #2, and #9) had integrated more physical education (PE) within schedules, having four or six weekly lessons of PE compared to the norm of two lessons. Scheduling PA/PE was done to support teachers for whom PA did not naturally lend itself to the curriculum: *“I think that planning PA into schedules helps. Well, it helps to know that if you forget all about PA, you at least have a lesson dedicated to PA”.* (Principal, school #4).

Five schools (school #1, #2, #7, #8, and #9) developed principles, meeting structures, or conducted workshops in order to achieve motivation for delivering PA within the curriculum, intending to heighten school staff commitment. These initiatives were all developed through shared decision-making between school managers and teachers, ensuring that the school developed a shared vision for how to deliver PA within the curriculum: *“I think it’s about developing a culture … a shared vision … and a common mindset about the importance of PA”.* (Deputy principal, school #7).

Seven schools collaborated with external parties as part of their PA implementation. Two schools (school #2 and #6) collaborated with the municipality or a national sports organization, participating in PA promotion projects. Five schools (school #2, #4, #6, #8, and #9) had established collaborations with local sports associations, inviting sports associations to organize workshops for the students or take part in PE lessons. All school managers collaborating with external parties found it beneficial in order to implement PA within the curriculum:

*“We have established a collaboration with an athletics club and a cycling club. ( … ) We have some facilities and opportunities here at the school, but the collaborations are also about being able to thrive on associations with other facilities and opportunities than what we have here”.* (Principal, school #4)

Thus, collaboration with external parties helped schools accomplishing the mandatory PA component, putting facilities and instructors at disposal.

#### Maintenance

From the interviews, four key findings for maintenance of PA within the curriculum were found: internal coordinators, motivated school staff, school management priority, and municipal support. Six school managers (school #2, #3, #4, #5, #9, and #10) stated that internal coordination was essential for maintenance. The coordinators were, among other things, important for sharing hands-on knowledge and inspiring teachers to deliver PA in new and different ways:

*“In order to continue delivering PA within the curriculum, we will continue having PA coordinators at all bases [year groups, Ed.]. And if one of those leave another one will take its place”.* (Deputy principal, School #9)

Having teachers especially motivated for PA was another important factor for maintenance. Two schools (school #2 and #9) even stated that, when hiring, they searched for teachers interested in PA. In order to ensure maintenance, several principals also stated that PA needed to be a school management priority, allocating resources for PA education and materials, and leading a common strategy for delivering PA within the curriculum: *“I think there are things that are essential for sustainability. One such thing is that it has to be a management priority”.* (Deputy principal, School #2).

Interviews showed, however, that school managers felt a lack of support from the municipality to maintain PA initiatives within the curriculum. Local politics were perceived to change and evolve continuously, and schools were obligated to support policy developments on a huge number of areas — PA being just one of these. At times, the sheer volume of new initiatives made it difficult for school managers to dedicate adequate resources for one area like PA:

*“In our municipality, we are required to produce something called ‘focus areas’. I think I am about to produce the sixth focus area within one and a half year. And if you continue to introduce a new focus area every two or three months, you will lose sight of the focus area you were using 9 months ago”* (Principal, school #3).

## Discussion

The objective of the current study was to explore the reach, effectiveness, adoption, implementation, and maintenance of mandatory PA within the curriculum at ten Danish schools. The RE-AIM evaluation tool identified central factors discussed further below.

### Commitment and school culture

Generally, school managers and teachers in the sample were interested in the PA requirement, finding it meaningful, believing that PA within the curriculum was important, and that PA could advance student learning. However, these findings do not necessarily reflect teachers’ willingness to actually implement PA within the curriculum. For instance, at the two schools with the lowest percentage of students reaching the requirement, all teachers responding to the questionnaire agreed on the aforementioned factors. Thus, the attitude toward PA might be removed from the actual behavior at these schools. Despite this finding, interest in PA has been reflected by others as an advantage for the implementation process, since it ensures that both teachers and school managers already having an awareness of the importance of the requirement [12, 13, 15].

Forty percent of the schools in the sample had already adopted PA as a central part of the school culture prior to introduction of the school reform. Three of those schools had the highest percentage of students reaching the PA requirement measured by accelerometry. The extent to which the requirement fits within an organization’s mission, priorities, and values has previously been pointed out as impacting the commitment towards realizing the implementation [12, 13, 29, 30]. A study by Webster et al. (2020) found, however, that most schools are largely unprepared to implement multicomponent approaches (e.g., the Danish school reform), inhibiting schools from adopting the program. Thus, existing school culture might be an important factor for the degree of implementation.

### Organisation of PA within the curriculum

Almost two-thirds of the teachers reported delivering PA within the curriculum weekly (53.4%), but the number reporting daily delivery, in keeping with the requirement, was extremely low; less than 10%. In concert with this, less than half of the students achieved 45 min of daily PA. Thus, the requirement was initiated to some degree, but was far from fully implemented.

Two key suggestions related to implementation of the mandatory PA within the curriculum were highlighted by the school managers: scheduling PA within school schedules and collaborations with external parties. Introducing a mandatory PA requirement strongly urges school staff to adhere to the requirement. At the same time, there has been an increasing pressure placed on teachers to improve academic performance, and some teachers perceive time spent on academic work to be more beneficial compared with time spent on PA [31]. Prioritizing becomes even more strained. Thus, planning PA/PE within school schedules helps ensure that students achieve the mandatory amount of PA within the curriculum, while teachers can focusing on the content of the academic teaching in the remaining lessons. Sixty percent of the schools in the sample scheduled their PA in the daily schedule. At three of those schools, more than 70% of the students achieved the requirement. Two of these schools had tripled the amount of weekly PE and the last one had scheduled 30 min dedicated to PA daily. At the remaining three schools, 30–40% of students reached the requirement. For some schools, scheduling PA/PE seemed beneficial in order to accomplish the daily mandatory PA within the curriculum. This is also reflected in previous studies, highlighting scheduling of PA as a facilitator to implementation of PA policies in schools [32]. Moreover, well-defined program components and an extensive teacher manual to support implementation has also been regarded as important for program implementation [12, 33].

Another way to help schools to accomplish the PA requirement could be through collaborations with external parties (e.g., local or national sports clubs or consultant or instructor from the municipality). This is in line with the Comprehensive School Physical Activity Program (CSPAP) Model, including family and community engagement as one part of the model. This model endorses engagement of families and the community in school events to increase students PA levels [34]. This is also reflected in other studies, showing that cooperation and collaboration among local agencies (e.g., partnerships, networking) are beneficial, bringing different perspectives, skills, and resources to bear on the implementation [13, 35, 36]. A study by de Meij et al. (2013) further supports collaborating with external parties, stating that involvement and support of experts in sports, health, and education is a facilitating factor for implementation at the user level.

### Motivation of school staff

Most school managers in the sample had experienced teachers who were poorly prepared for handling PA within the curriculum, highlighting the importance of competencies development. While often defined as development of skills necessary for implementation, competencies development is equally about having a fundamental mindset about how to handle the implementation [30, 35, 37]. In addition, Durlak and Dupre (2008) state that development of competencies is also about developing motivation and self-efficacy. Such qualities affect future performance [13]. Thus, motivated school staff is an important factor for the implementation process and has been identified to affect both adoption, implementation, and maintenance [12, 38]. This is in line with Nielsen et al. (2018), highlighting the need for teachers to be trained to develop the skills and self-efficacy needed to feel motivated and dedicated to the implementation. However, despite receiving training and education, many teachers have relatively little knowledge and skills in relation to implementation of PA [39]. Moreover, despite having received training and education, some teachers may still opt not to accommodate the implementation because they are busy with other duties within an educational system where academic performance is the number one priority [34].

In order to ensure motivated school staff, internal coordinators were highlighted as favorable agents to include in the implementation process, taking care of workshops, competencies development, and ongoing training for teachers. One of the five schools, which stated that internal coordinators were essential for maintenance, was the school with highest effectiveness. This school had a team of coordinators, mostly consisting of PE teachers, responsible for making a common thread for integration of PA, ensuring that all teachers, independent of PA competencies, were able to deliver qualified PA within the curriculum. At the four other schools that supported the use of internal coordinators, between 30 and 38% of the students were active during curricular time. However, the schools in question were just embarking on the process of organizing internal coordinators. Appointing internal coordinators or program champions has been highlighted as being advantageous to ensure a successful implementation process [13, 29, 40]. Program champions, particularly senior staff within an organization and who are respected by the other staff, can do much to help orchestrate a program through the entire diffusion process from adoption to maintenance [13]. This could be a school manager, but it could also be PE teachers, classroom teachers, administrators, or others who are well-suited to lead the implementation [34, 41].

### School management support

The support of school management was shown to be vitally important for developing a school culture prioritizing the delivery of PA within the curriculum. This is also highlighted by others, reporting that school management support is a clear enhancement in securing motivation to PA programs [12, 13, 33]. Moreover, in a school-based mental health program, Kam et al. (2003) showed a significant association between school management support and teachers’ fidelity of the implementation on student outcomes. Students improved significantly on all outcomes when both school management support and teachers’ fidelity of the implementation were high. However, this research found several negative changes when school management support was low. This underlines the importance of school management support of the program [42].

School managers in the sample had experienced teachers that were skeptical towards the mandatory PA components, which could be due to the additional workload following the requirement. A study on the implementation of the CSPAP found that many schools view the addition of PA program as an extra responsibility in an already over-loaded school agenda [34]. Other studies show that when introducing new programs, teachers are concerned about additional workloads which challenge them to prioritize possibilities and obligations [15, 43, 44]. Our study highlights the importance of shared decision-making, as teacher involvement reportedly heightened commitment to the program. Involvement of teachers in decision-making processes (e.g., development of a PA implementation strategy) has previously been highlighted as a cornerstone in implementation of PA programs, uniting organizational members regarding the value and purpose of the program [12, 45]. This further clarifies the importance of school management support, taking responsibility for leading the implementation process through teacher involvement and dialog in order to ensure motivation and ownership for the implementation.

Our research suggests that school management support of PA implementation would be a central factor in maintenance. This is in line with previous research, highlighting that school management support is crucial for creating coherence and prioritizing in situations where consensus about what exactly should be done many times is only partial [13, 35, 38, 46]. A review by Cassar et al. (2019), among others, also found that active involvement of school managers, supporting and prioritizing the PA program, was a key determinant for both implementation and maintenance [12, 47–49].

### Municipal support

Municipal support was addressed as an important factor for maintenance. However, some schools felt a lack of municipal support during the implementation of the PA requirement. One of those schools was the school with the lowest percentage of students reaching the PA requirement.

Schools and the educational system are busy with the core business of teaching and learning. This premise affects specific agendas on, for instance, increasing the volume of curriculum-based PA [50]. The need for continued municipal support is frequently highlighted — stressing that allocation of resources to schools (e.g., time for schools to develop an PA strategy or support regarding facilities) is needed [13, 51, 52]. Moreover, a study by Skovgaard & Johansen (2020) highlighted the importance of managers (both school and district managers), employees, and other core stakeholders developing a shared strategy for the area and setting ambitious goals that can realistically be achieved. Such a strategy could help both schools and municipalities to reach a common understanding of the implementation process – that it takes time and that schools are not able to implement new strategies every two months.

The two schools with the highest percentage of students reaching the PA requirement were located in the same municipality. All schools in this municipality were financially supported by the municipality if they chose to become part of their local PA program, tripling the amount of weekly PE. This case is an example of a successful implementation process with active involvement of both schools and municipalities in developing a shared strategy [19].

## Methodological considerations

A strength of this study was the use of multiple data sources including accelerometers, a questionnaire, and interviews, as it provided a more comprehensive understanding of the RE-AIM dimensions and strengthened the external validity, credibility, and transferability of the study [22, 53]. That being said, we do recognize that this study has some limitations. Generally, eight of the included schools were located in the region of Southern Denmark and two schools were located in the capital region. Inclusion of schools from other regions would have been beneficial, ensuring greater national representativeness. Due to the design of the PHASAR study, this was not a possibility, though [21].

Another general limitation was the inclusion of students from different grades, which made it difficult to compare schools. Consequently, the results indicate that students in lower grades are more active than students in upper grades. Ideally, students from the same grades should have been included at all of the participating schools. Unfortunately, this was not possible due to the PHASAR study design. Although some steps were taken to achieve a comparable sample, the age factor still seemed to be of considerable importance to student PA levels.

Another limitation is that recall bias may have emerged, since the interviews were conducted three to four years after introduction of the school reform. Finally, it is important to acknowledge that the present study only represents teachers’ views through the questionnaire. The teachers were unfortunately not able to take part in the interviews due to limited time allocated for taking part in the study.

## Conclusions

Using the RE-AIM framework, this study explores the implementation of a nationwide requirement mandating integration of daily PA into Danish public school curricular time. Notably, implementation varied across schools and could be described as partially implemented after four years. Our study showed the importance of school management in monitoring, prioritizing, and supporting the implementation process and taking the lead on establishing a school culture centered around PA. We also showed the importance of teacher involvement, ensuring motivation and ownership for the implementation.

In terms of implementation, the benefit of appointing an internal coordinator or coordination teams to provide teacher competence development; inspirational materials easy for teachers to use within the curriculum; and ongoing training were highlighted. Finally, it seems beneficial for municipalities, school managers, teachers, and other stakeholders to develop a shared strategy for the implementation process and to set ambitious goals, which are realistic to achieve. Thus, targeting the implementation at multiple levels within the educational system, such as the capacity of school managers to lead the process, teacher buy-in by active involvement, internal coordinators, and municipal support, might increase the probability for successful implementation of scaled-up real-world programs.

## Data Availability

The dataset supporting the conclusions of this article are available at the time of publication upon application to the PHASAR Steering Committee (ktlarsen@health.sdu.dk). If approved by the Steering Committee and the Danish Data Protection Agency, data will be available.

## Abbreviations

PA: physical activity
PE: physical education
RE-AIM: Reach, Effectiveness, Adoption, Implementation and Maintenance
PHASAR: Physical Activity in Schools After the Reform
Redcap: Research Electronic Data Capture
PRISM: Practical, Robust Implementation and Sustainability Model
